# Proteomic Profiling of Saliva and Tears in Radiated Head and Neck Cancer Patients as Compared to Primary Sjögren’s Syndrome Patients

**DOI:** 10.3390/ijms23073714

**Published:** 2022-03-28

**Authors:** Håvard Hynne, Lara A. Aqrawi, Janicke Liaaen Jensen, Bernd Thiede, Øyvind Palm, Cecilie Delphin Amdal, Kristine Løken Westgaard, Bente Brokstad Herlofson, Tor P. Utheim, Hilde Kanli Galtung

**Affiliations:** 1 Department of Oral Surgery and Oral Medicine, Faculty of Dentistry, University of Oslo, 0317 Oslo, Norway; havard.hynne@odont.uio.no (H.H.); laraadnan.aqrawi@kristiania.no (L.A.A.); j.c.l.jensen@odont.uio.no (J.L.J.); k.l.westgaard@odont.uio.no (K.L.W.); b.b.herlofson@odont.uio.no (B.B.H.); 2 Department of Health Sciences, Kristiania University College, 0153 Oslo, Norway; 3 Department of Biosciences, University of Oslo, 0371 Oslo, Norway; bernd.thiede@ibv.uio.no; 4 Department of Rheumatology, Oslo University Hospital, 0372 Oslo, Norway; oypalm@gmail.com; 5 Section for Head and Neck Oncology, Oslo University Hospital, 0372 Oslo, Norway; cecia@ous-hf.no; 6 Department of Otorhinolaryngology—Head and Neck Surgery Division for Head, Neck and Reconstructive Surgery, Oslo University Hospital, 0372 Oslo, Norway; 7 Department of Medical Biochemistry, Oslo University Hospital, 0372 Oslo, Norway; utheim2@gmail.com; 8 Institute of Oral Biology, Faculty of Dentistry, University of Oslo, 0316 Oslo, Norway

**Keywords:** radiotherapy, head-and-neck cancer, Sjögren’s syndrome, saliva, tear fluid, salivary glands, lacrimal glands, meibomian glands, proteomics, immune response, inflammation, tissue healing, biomarkers

## Abstract

Patients with head and neck cancer (HNC) and patients with primary Sjögren’s syndrome (pSS) may exhibit similar symptoms of dry mouth and dry eyes, as a result of radiotherapy (RT) or a consequence of disease progression. To identify the proteins that may serve as promising disease biomarkers, we analysed saliva and tears from 29 radiated HNC patients and 21 healthy controls, and saliva from 14 pSS patients by mass spectrometry-based proteomics. The study revealed several upregulated, and in some instances overlapping, proteins in the two patient groups. Histone H1.4 and neutrophil collagenase were upregulated in whole saliva of both patient groups, while caspase-14, histone H4, and protein S100-A9 were upregulated in HNC saliva only. In HCN tear fluid, the most highly upregulated protein was mucin-like protein 1. These overexpressed proteins in saliva and tears play central roles in inflammation, host cell injury, activation of reactive oxygen species, and tissue repair. In conclusion, the similarities and differences in overexpressed proteins detected in saliva from HNC and pSS patients may contribute to the overall understanding of the different pathophysiological mechanisms inducing dry mouth. Thus, the recurring proteins identified could possibly serve as future promising biomarkers

## 1. Introduction

Head and neck cancer (HNC) is the sixth most common cancer in the world [1], and constitutes a group of cancers located in the oral cavity, larynx, pharynx, sino-nasal cavities, and salivary glands [2]. Among these, oral- and oropharyngeal cancers are the most prevalent, and squamous cell carcinoma represents more than 90% of the cases [3]. Radiotherapy (RT) is often used to treat HNC, either alone or in combination with surgery and chemotherapy [4], and intensity-modulated radiotherapy (IMRT) is now often applied to maximise delivery to the targeted tissue [5] and reduce normal tissue toxicity [6]. Nevertheless, RT may still induce adjacent normal tissue damage, such as impairment of the salivary and lacrimal gland function [7,8], where higher radiation doses, targeted tissue volume, and tumour localisation are additional contributing factors [9,10].

Hallmarks of primary Sjögren’s syndrome (pSS) are reduced salivary and lacrimal gland function [11,12], most likely due to autoantibody production and mononuclear cell infiltration in these disease target organs, resulting in reduced secretion of tears and saliva [13]. Hence, both pSS patients and patients treated for HNC may exhibit symptoms of dry mouth and dry eyes, although the cause of their symptoms is dissimilar.

To date, the etio-pathological mechanisms associated with ocular and oral dryness are still not fully understood. Studying the proteome of biological fluids and screening for disease-specific biomarkers through liquid chromatography–mass spectrometry (LC-MS) has, therefore, been in focus over the last decades [14]. Indeed, the proteomes of both saliva [15,16,17] and plasma [4,18] have previously been investigated in HNC patients to study the effects of RT, and late effects have also been considered in saliva protein profiles [19]. Consequently, intercellular signalling proteins that may play a role in regulating cell growth, cellular proliferation, angiogenesis, tissue repair, and immune responses to infection, injury, and inflammation could be identified [20]. We have already indications that biofluids such as saliva and tears may contain valuable biomarkers for diagnostic and therapeutic purposes [14]. Sampling of saliva as compared to blood and tissue is favourable in many ways, such as undemanding collection, non-invasiveness, and easy shipment and storage of samples. This has led to the development of devices and technologies for detection of biomarkers in saliva ranging from identification and quantification of viral nucleic acids during the coronavirus disease 2019 pandemic to monitoring drug abuse.

We have previously investigated the proteome of saliva and tear fluid in pSS through LC-MS [14,21], and the salivary and lacrimal cytokine profiles in pSS and in radiated HNC patients through multiplex bead-based immunoassays [22,23]. Indeed, it is of interest to compare the salivary and tear proteome of these radiated patients to that of pSS patients, since the former group may also display symptoms of dry eyes and dry mouth, and possibly also show mild signs of inflammation as a consequence of the localised RT administered [23]. In the present study, we investigated the proteome of saliva and tear fluid of radiated HNC patients in the same individuals through LC-MS at least six months post radiation treatment. The purpose of the study was to establish a better understanding of the pathophysiology and biochemical processes behind dry mouth and dry eye disease, and to gain more insight into the biochemical composition of saliva and tear fluid. Moreover, by comparing two different patient groups suffering from dry mouth, we sought to identify the biochemical pathways that can be used to discriminate between patient groups and provide targets for further analyses of the mechanisms. Our aim was to investigate the late effects of RT on protein expression and cellular pathways in radiated HNC patients. A further aim was to explore how these alterations compare to protein expression patterns in patients with pSS and in healthy controls. We conclude that by studying the late effects of RT through proteomic profiling in saliva and tears in HNC, and comparing these findings to those in pSS, we could identify proteins that may serve as promising disease biomarkers.

## 2. Results

### 2.1. Quantitative Proteomics Analysis of Whole Saliva

**Figure 1:**
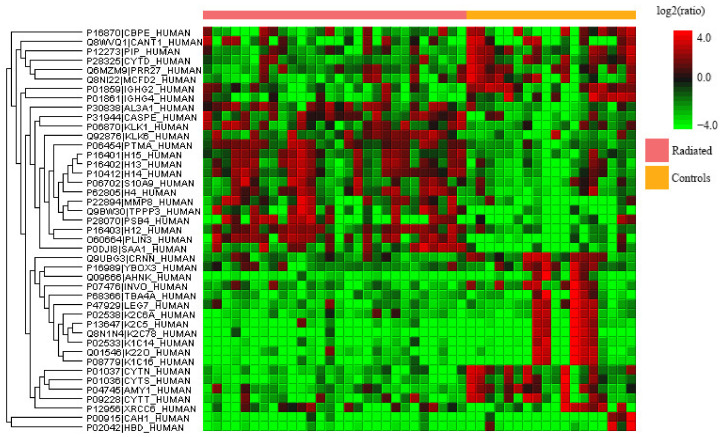
Heatmap of the over- (red) and under-expressed (green) proteins detected in whole saliva of radiated head and neck cancer patients vs. controls.

**Figure 2:**
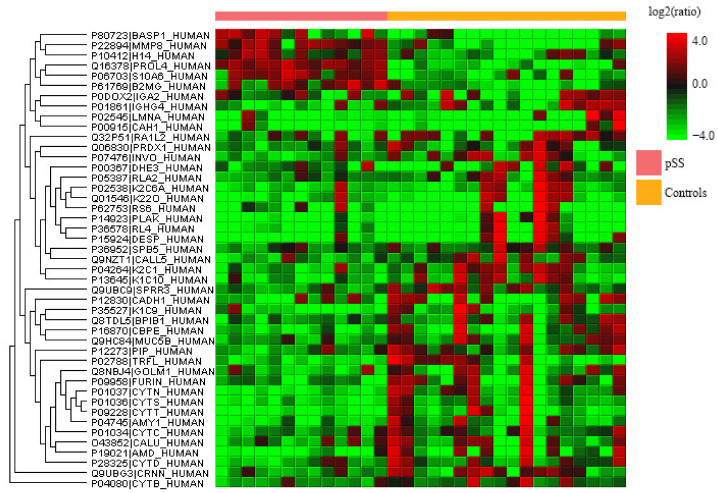
Heatmap of the over- (red) and under-expressed (green) proteins detected in whole saliva of primary Sjögren’s syndrome (pSS) patients vs. controls.

**Figure 3:**
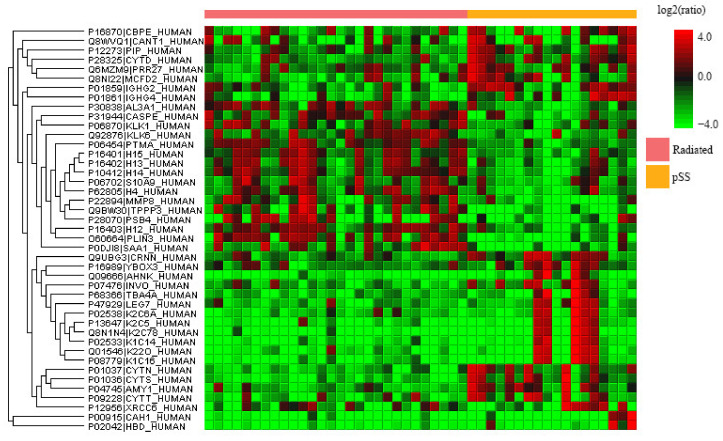
Heatmap of the over- (red) and under-expressed (green) proteins detected in whole saliva of radiated head and neck cancer patients vs. primary Sjögren’s syndrome patients (pSS).

Label-free quantitative proteomics was performed on whole saliva of radiated HNC patients, pSS patients, and healthy controls to find the up- and downregulated proteins between the different groups. The upregulated proteins are shown in Table 1 while the downregulated ones are found in Table 2. A few of the upregulated proteins were observed in two comparisons and are marked accordingly in the tables. Common proteins for the comparison of HNC and pSS against the controls, respectively, included histone H1.4 and neutrophil collagenase. Additionally, three upregulated proteins were common for both radiated HNC patients vs. controls and HNC patients vs. pSS (caspase-14, histone H4, and protein S100-A9). An overview of all the significantly up- and downregulated proteins in whole saliva, with the group comparisons expressed as ratios, is visualized in the heat maps shown Figure 1, Figure 2 and Figure 3. Considering matching names, five histones (H1.2, H1.3, H1.4, H1.5, and H4) and four protein S100 (A6, A7, A8, and A9) were found in the list of upregulated proteins, and six cystatins (B, C, D, S, SA, and SN), and four immunoglobulins (three heavy gamma and one heavy alpha) for the downregulated proteins.

Functional annotation cluster analysis of the up- and downregulated protein sets was performed using Database for Annotation, Visualization and Integrated Discovery (DAVID) Bioinformatic resources. This analysis revealed only few terms with an enrichment score more than 3 (Table 3). Histones and certain proteases were found to be significantly upregulated and cystatins downregulated in HNC patients compared to healthy controls. Cystatins were also found to be downregulated comparing pSS patients against controls.

**Figure 4:**
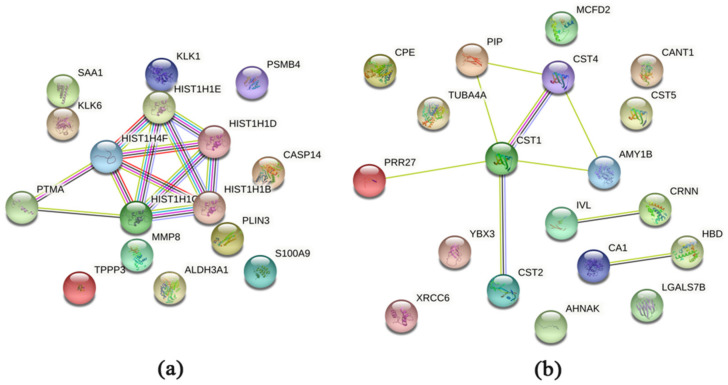
Protein–protein interactions of the significantly up- and downregulated proteins in saliva from HNC patients. The interaction map of the upregulated proteins is shown in panel (**a**) and the map of the downregulated proteins in panel (**b**). The Search Tool for the Retrieval of Interacting Genes/Proteins (http://string-db.org/ (accessed on 10 January 2022) was used to generate the networks, where potential interactions of proteins with medium confidence are shown. The colour of the connecting lines indicates the type of evidence used in predicting the associations (light blue: known interactions from curated databases; pink: known interactions experimentally determined red gene fusion; green: predicted interactions from gene neighbourhood; red: predicted interactions from gene fusions; dark blue: predicted interactions from gene co-occurrence; yellow/green: protein–protein associations through text-mining extracted from the literature; black: protein–protein associations through co-expression; light purple: protein–protein associations through protein homology).

The protein–protein interaction analysis of the regulated proteins was performed using STRING and revealed for the comparison of HNC against controls similar results as the functional annotation cluster analysis using DAVID (Figure 4). For the upregulated proteins in HNC, the five histones (Table 3) are central, in addition to prothymosin alpha (PTMA) (Figure 4a). For the downregulated proteins, the cystatins (cystatin-SN (CST1), cystatin-SA (CST2), and cystatin-S (CST4)) appeared together, including alpha-amylase 1b (AMY1B), prolactin-inducible protein (PIP), and proline-rich protein 27 (PRR27) (Figure 4b).

### 2.2. Quantitative Proteomics Analysis of Tear Fluid

**Figure 5:**
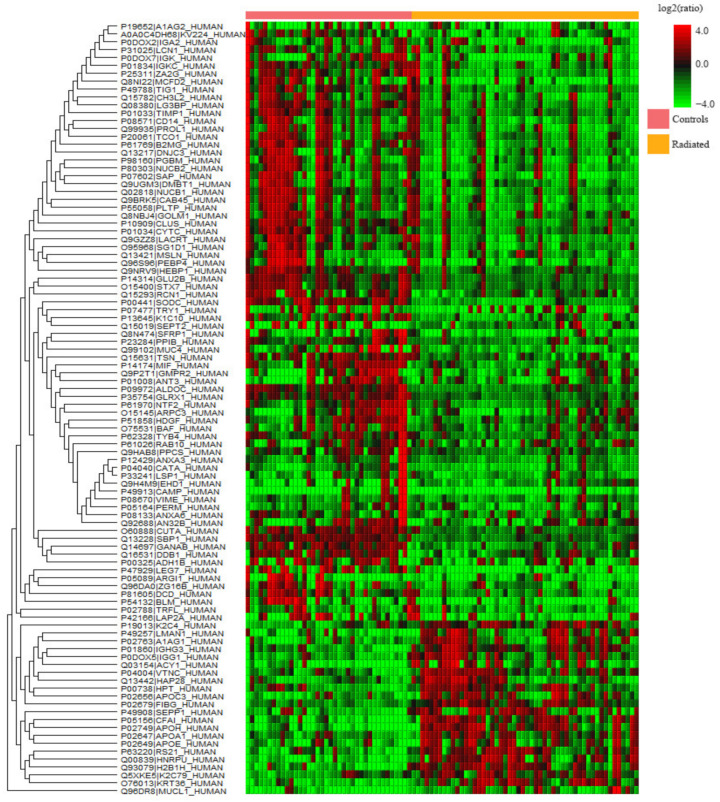
Heat map of the over- (red) and under-expressed (green) proteins detected in tear fluid of radiated head and neck cancer patients compared to the controls.

Label-free quantitative proteomics was performed on tear fluid of radiated HNC patients and healthy controls to find the up- and downregulated proteins. The upregulated proteins are shown in Table 4 while the downregulated ones are found in Table 5. An overview of all the over- and under-expressed proteins detected in the tear fluid of the radiated patients compared to the controls is visualized as a heat map in Figure 5. Considering matching names, four apolipoproteins (APOA1, APOC1, APOE, and APOH) were found in the list of upregulated proteins, and six cystatins (B, C, D, S, SA, and SN) and four immunoglobulins (three kappa and one alpha) for the downregulated proteins.

Functional annotation cluster analysis of the up- and downregulated protein sets was performed using DAVID Bioinformatic resources. The functional cluster analysis revealed only few terms with an enrichment score more than 3 (Table 6). Secreted/extracellular and lipid-binding proteins (both groups containing four apolipoproteins) were found to be significantly upregulated while the EF-hand domain was found to be downregulated in HNC patients compared to healthy controls.

**Figure 6:**
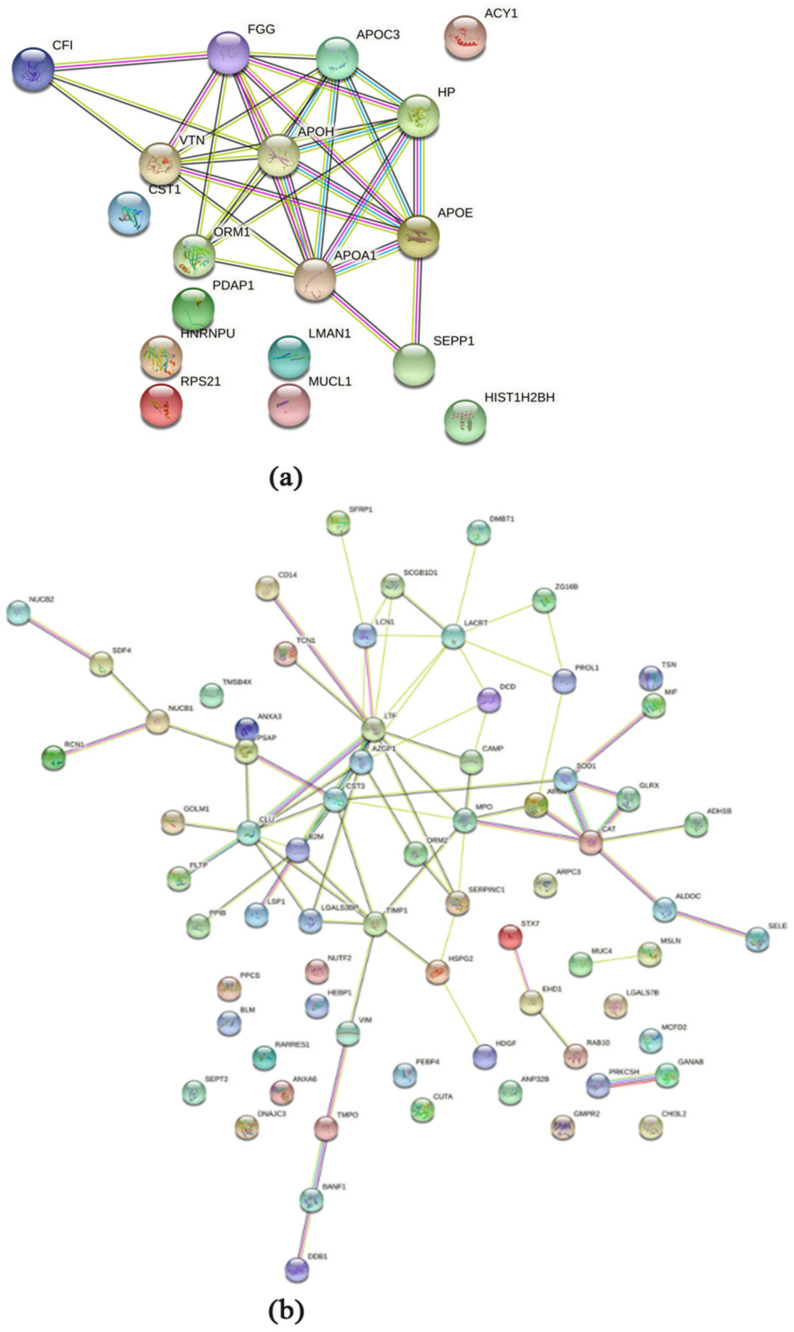
Protein-protein interactions of significantly up- and downregulated proteins in tear fluid from HNC patients. The interaction map of upregulated proteins is shown in panel (**a**), and the map of downregulated proteins in panel (**b**). The Search Tool for the Retrieval of Interacting Genes/Proteins (http://string-db.org (accessed on 10 January 2022)) was used to generate the networks, where potential interactions of proteins with medium confidence are shown. The colour of the connecting lines indicates the type of evidence used in predicting the associations (light blue: known interactions from curated databases, pink: known interactions experimentally determined red gene fusion, green: predicted interactions from gene neighbourhood, red: predicted interactions from gene fusions, dark blue: predicted interactions from gene co-occurrence, yellow/green: protein-protein associations through text-mining extracted from literature, black: protein-protein associations through co-expression, light purple: protein-protein associations through protein homology).

A protein–protein interaction analysis of the regulated proteins was performed using STRING and revealed for the comparison of HNC against the controls similar results as the functional annotation cluster analysis using DAVID (Figure 6). For the upregulated proteins in HNC, the apoplipoproteins are building a group together with fibrinogen gamma chain (FGG), alpha-1 acid glycoprotein 1 (ORM1), selenoprotein P (SEPP1), and vitronectin (VTN) (Figure 6a). For downregulated proteins, the interaction map appears more scattered (Figure 6b).

### 2.3. Pathway and Biological Processes Analysis of Saliva and Tear Material Using DAVID and FunRich

When comparing the proteins detected in whole saliva from radiated HNC patients or pSS patients to controls using DAVID we found enriched pathways that included regulation of salivary secretion (*p* < 0.01 for both patient groups; results not shown). Inspecting the list of genes involved in these pathways, we observed the proteins cystatin D, S, SA, and SN in both patient groups. Additionally, cystatin C, calmodulin like 5 and mucin 5B were found in the pSS patients.

**Figure 7:**
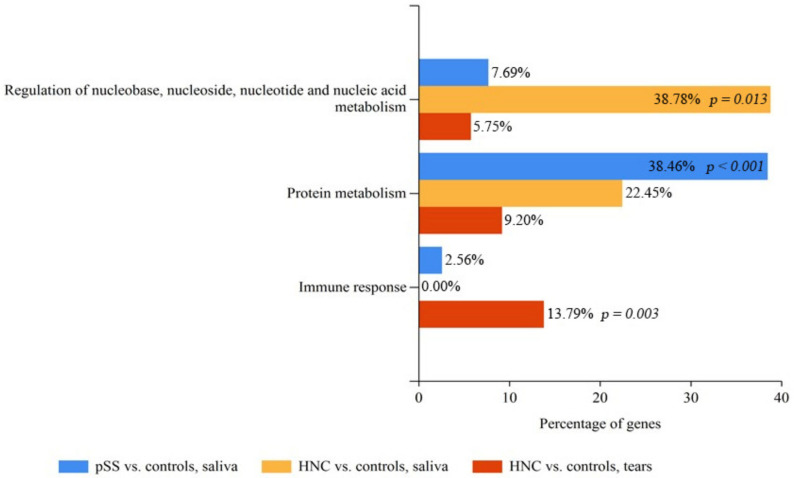
FunRich analysis delineating the up- and downregulated biological processes identified in patients radiated for head and neck cancer (HNC) when compared to the controls, and patients with primary Sjögren’s syndrome (pSS) when compared to controls. Biological processes were identified using FunRich database and FunRich version 3.1.3 (2017).

FunRich analysis of the biological processes on the same three groups identified a diversity in the biological processes up- or down regulated (Figure 7; biological processes with statistical significance in one of the groups were included). In HNC patients compared to controls “Regulation of nucleobase, nucleoside, nucleotide and nucleic acid metabolism” was significantly upregulated in saliva (genes involved: H1-4, H1-2, H4C1, H4C1, H1-3, H1-5, YBX3, XRCC6; *p* = 0.013) while “Immune response” was significantly increased in tear fluid (genes involved: AZGP1, B2M, CAMP, CD14, CFI, CLU, DMBT1, HP, LGALS3BP, MSLN, ORM1, ORM2; *p* = 0.003). For pSS patients versus controls, “Protein metabolism” was significantly upregulated (genes involved: MMP8, SERPINB5, RPLP2, CSTB, CST3, CRNN, CST5, FURIN, CPE, RPS6, PAM, CST4, CST1, RPL4, CST2; *p* < 0.001).

## 3. Discussion

The present study is the first to explore proteome profiles simultaneously in saliva and tear fluid in patients with HNC post-RT. Furthermore, we compared these results to protein expression of saliva in pSS patients, as impaired saliva and tear production are common occurrences in these patient groups, but the pathogenesis is not well understood. We identified both up- and downregulated signalling pathways and proteins that, to the best of our knowledge, have not been reported previously. Signalling pathways and proteins common to both groups were also identified.

Enrichment analysis/gene ontology term analysis of salivary proteins using the DAVID software revealed cellular pathways that regulate salivary secretion in both patient groups. The radiated HNC patients in this study received on average a 13 times higher radiation dose to the parotid glands (mean 23.1 ± 10.2 Gy, range 1.6 to 48.5) as compared to the lacrimal glands (mean 1.8 ± 4.2 Gy, range 0.3 to 17.5). Notably, more protruding oral manifestations as compared to ocular findings have previously been reported in these patients [10]. Moreover, radiation doses above 15–20 Gy, that target a larger tissue volume could contribute to more severe damage to the salivary glands [7], which may in turn trigger both inflammation and tissue repair mechanisms.

Interestingly, we found that saliva in HNC patients demonstrated upregulated levels of serum amyloid A-1, while this protein was not found in tear fluid. The serum amyloid proteins have powerful pro-inflammatory and cytokine-like properties, and have been found to be highly expressed in a number of malignancies [24,25]. Thus, this protein could play a role in the acute and late effects of RT, and the upregulated levels in saliva as compared to tears may be a consequence of the lower radiation dose received by the lacrimal glands.

Tumour pathogenesis may indeed also be viewed as an autoimmune reaction [26,27,28], where immune responses may lead to tissue damage [29,30,31] followed by wound repair [32]. Moreover, tissue healing may also be triggered in the radiated patients as a consequence of the tissue damage [33,34] and immune alterations [35] caused by the RT administered. On a similar note, the activities of tissue healing and immune alterations mentioned above could be related to the observed upregulated biological processes “Immune response” and “Regulation of nucleobase, nucleoside, nucleotide and nucleic acid metabolism” found with FunRich analysis. The former as part of the inflammation response and the latter as a component of the tissue regeneration process [36]. Furthermore, protein–protein interactions, as visualized with STRING analysis, involving several histones, could certainly point to a regulation of replication and transcription that one would expect during tissue healing. These processes are most likely upregulated in tissue remodelling following radiation treatment [33,34]. The linker histone H1 is of particular interest as it also seems to be implicated in diseases such as cancer [37,38].

The proteomic profiling performed revealed several upregulated, and in some instances overlapping, proteins in whole saliva in the two patient groups. In whole saliva, both groups expressed upregulation of histone H1.4 and neutrophil collagenase. In supplement to their functions mentioned above, histones can trigger inflammatory responses, and have even been shown to induce host cell injury under certain circumstances [39]. In vitro studies have demonstrated that depletions of either H1-2 or H1-4 strongly reduce the ability of neutrophils to form neutrophil extracellular traps [40]. Interestingly, neutrophil extracellular traps contain neutrophil collagenase. Expression of neutrophil collagenase has been found to be protective in human squamous cell carcinoma of the tongue and is regarded as tumour- or metastasis suppressive [41]. Additionally, animal models indicate that neutrophil collagenase may have a protecting effect in autoimmune disorders [42]. Clusters of downregulated proteins in saliva of both HNC and pSS patients included several cystatins, amylase, and a proline-rich protein. As these proteins are all part of the basic and enriched repertoire of the salivary proteome, these findings are in accordance with the effects of reduced salivary gland function. In terms of clinical relevance, histone modification has previously been suggested as a new treatment in pSS [43], and the results from the present study indicate that they may play a role in other dry mouth conditions as well. Furthermore, cystatins have been reported to be in lower concentrations in subjects with xerostomia compared to subjects with similar salivary flow rates [44]. The current study suggests that this should be explored further, and may help in understanding the lack of correlation between symptoms and objective findings in dry mouth [45].

Several proteins were upregulated in saliva of HNC patients as compared to both pSS and controls. Of these, histone H4, protein S100-A9, and caspase-14 are of interest. The expression of the S100-A9 gene has been found to be regulated in response to irradiation in a mouse model [46]. Although here the protein was downregulated, this could indicate that S100A9 is responsive to RT. Interestingly, it has been observed that salivary S100-A9, in an S100-A8/S100-A9 complex, is significantly increased in pSS patients at risk of developing lymphoma [47]. The authors suggested that this finding may be related to the potential role of S100-A9 as an amplifier of inflammation-associated tumour development. Finally, caspase-14 is a non-apoptotic protein associated with the epidermis, where it plays a role in keratinocyte differentiation [48]. There are, however, indications that it could be involved in tumour suppression [49], although its role in HNC patients following RT is yet to be elucidated.

Even though the eyes and the lacrimal glands received a relatively low radiation dose compared to the salivary glands, we found changes in the protein profile of the tear fluid in HNC patients as compared to healthy controls. The most highly upregulated protein was mucin-like protein 1. This protein is associated with meibomian gland (MG) dysfunction [50]. MGs are found in the upper and lower lids of the eyes, and it is not unlikely that these glands were affected by the RT in our study. Indeed, in a previous study from our group we demonstrated functional and morphological changes in MGs of radiated HNC patients, while there were no such changes in the lacrimal glands [51]. The MG affliction may predispose the patients to dry eye disease. Indeed, RT-treated HNC patients have dry eye complaints [10]. Finally, it has been found that MUCL1 is upregulated in dry eye patients, possibly as a compensatory response [52].

Additionally, tear fluid from HNC patients demonstrated a notable upregulation of several apolipoproteins (apolipoprotein C-III, apolipoprotein A-1, and apolipoprotein E). These findings were corroborated by functional annotation cluster analysis, where these proteins were delegated to the enriched terms “secreted/extracellular”. These findings are in line with Wildlak et al., who reported an RT-induced initial downregulation followed by upregulation of serum levels of apolipoprotein A-1, apolipoprotein A-2, apolipoprotein C-1, apolipoprotein C-2, apolipoprotein C-3, apolipoprotein L-1, and apolipoprotein M [53]. The reason for this increase is not apparent; however, apolipoprotein C-III has been found both to induce inflammation as well as activation of reactive oxygen species [54]. Thus, this upregulation could be a response to RT late effects. On the other hand, apolipoprotein A-1 has been found to have anti-oxidant effects [55]. Thus, the upregulation of this protein may be a cellular response to the observed post-irradiated oxidation [55].

Interestingly, proteins in the EF-hand domain were found downregulated in tear fluid from the HNC group compared to the healthy controls. This group of proteins is prominently known to be involved in Ca^2+^-signalling. The reason for why this group of proteins should be downregulated in the radiated HNC group is not evident, but could perhaps be related to disturbed Ca^2+^-induced signalling of tear fluid secretion in compromised lacrimal glands [56].

In conclusion, we found that overexpressed proteins in whole saliva and tear fluid play central roles in inflammation, host cell injury, activation of reactive oxygen species, and tissue repair in patients radiated for HNC, leading to the upregulation of interconnected cellular pathways in these individuals. Despite the radiated patient group being somewhat heterogenous, encompassing subjects who had received primary or adjuvant RT, with or without chemotherapy, we observed that cellular pathways that control salivary secretion were influenced both in patients radiated for HNC and in pSS subjects. The similarities and differences in the overexpressed proteins detected in saliva from HNC and pSS patients probably reflect the different pathophysiological mechanisms in autoimmunity in pSS and late effects of RT in HNC. Therefore, these findings may contribute to the overall understanding of the different pathophysiological mechanisms inducing dry mouth. This understanding may provide knowledge on what biochemical features are related to the pathological processes and what features are caused by the hyposalivation per se.

Since our findings are more explorative than directly clinically applicable, the results need to be validated in larger studies with longer follow-up in patients over time. Nonetheless, prior to clinical implementation, an understanding of the pathophysiological and biochemical processes and the correlations between salivary analytes and systemic changes is needed. For dry mouth and dry eye disease, such an understanding has not yet been achieved. A possible future approach is to utilize technologies such as machine learning or artificial intelligence allowing for data from a large number of variables, both clinical and biochemical, to be refined into clinically relevant information. Nonetheless, the recurrent proteins identified in the present study could serve as promising biomarkers when evaluating the late effects of RT in HNC. Future investigations are necessary both to validate these potential biomarkers in larger patient cohorts and to study their cellular roles in detail.

## 4. Materials and Methods

### 4.1. Study Population

**Figure 8:**
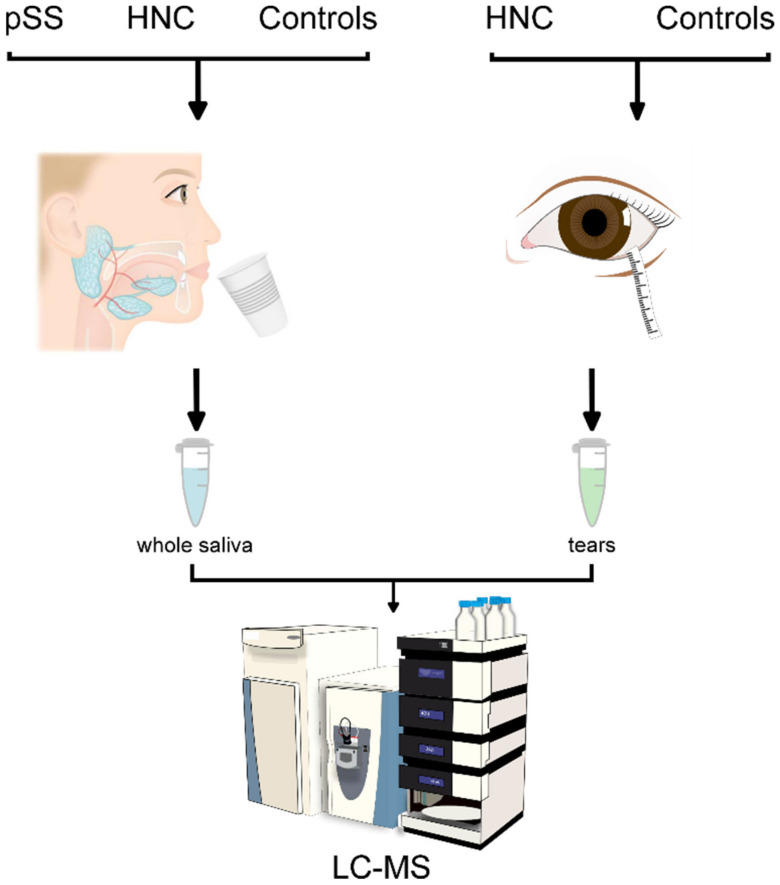
Graphical description of the study design. pSS: primary Sjögren’s syndrome patients; HNC: head and neck cancer patients; LC-MS: liquid chromatography–mass spectrometry. Copyright Emily Moschowits.

The participants included 29 patients diagnosed with HNC who had completed IMRT at least 6 months prior to recruitment, 14 pSS patients that fulfilled the American-European Consensus Criteria from 2002 [57], and 21 age- and sex-matched healthy individuals with no previous complaints of dry mouth or dry eyes. The HNC patients were recruited from the Department of Oncology, Oslo University Hospital, in the period September 2018 to March 2019. The pSS subjects were recruited from the Department of Rheumatology, Oslo University Hospital, in the period September 2015 to February 2018. A detailed explanation of the study aims and protocols were introduced to the subjects upon enrolment. Following recruitment, the patients were referred to the Dry Mouth Clinic at the Institute of Clinical Dentistry, Faculty of Dentistry, University of Oslo, and the Norwegian Dry Eye Clinic, Oslo, for thorough examinations and sample collection, as described below (two patients did not undergo eye examinations). This study was performed in compliance with the tenets of the Declaration of Helsinki, written informed consent was obtained from all participants, and the Regional Medical Ethical Committee of South-East Norway approved the study (2015/363 and 2018/1313). Figure 8 presents a graphical description of the study design.

All patients treated for HNC had received RT at the Department of Oncology, Oslo University Hospital, Norway. Information about the disease and treatment were extracted from the patients’ charts and specific treatment plan, and the dose estimations presented are exact dosages. Fourteen patients had been treated with primary RT (total dose of 68–70 Gy), and 15 patients received postoperative RT (total dose of 50–66 Gy), as previously described [10,23]. The average radiation dose to the parotid gland was 23.1 ± 10.2 Gy (range, 1.6 to 48.5 Gy), and to the lacrimal gland 1.8 ± 4.2 Gy (range, 0.3 to 17.5 Gy), delivered as 2 Gy per fraction, and administered 5–6 times per week; also, concurrent chemotherapy or targeted therapy (cisplatin or cetuximab) was given to 12 patients as part of the primary treatment for stage III–IV disease, or as part of the post-operative treatment in cases where there was marginal or perinodal infiltration (Table 7). All HNC patients recruited reported on problems related to dry mouth.

The pSS patients’ medical records and clinical data were obtained from their patient charts and through clinical examination at the Department of Rheumatology, Oslo University Hospital. Information that had been collected during routine laboratory assessments was provided, including anti-Ro/SSA and anti-La/SSB (autoantibody positivity), and evaluation of ocular and oral dryness via saliva and tear secretion ability. Some residual secretory ability was required for inclusion in the study to enable sample collection (Table 8).

### 4.2. Whole Saliva and Tear Fluid Collection

Participants underwent a thorough oral examination at the Dry Mouth Clinic, and stimulated whole saliva was collected as described earlier [10,58]. Strict routines were employed to ensure standardisation of the method for saliva collection, since secretory ability has been shown to vary depending on the nature of the stimuli, the time of day, and storage. Saliva was collected between 10 a.m. and 2 p.m., and the protocol and equipment used for saliva collection and storage were identical for all participants. In brief, subjects were asked to not intake any food or drink at least 1 h before saliva collection. Following the oral examination, the participants were asked to chew on a paraffin block (Ivoclar Vivadent, Shaen, Lichtenstein), while saliva was collected on ice for 5 min, and then aliquoted and stored at −80 °C.

Additionally, the HNC patients and controls also underwent a thorough ocular surface examination, followed by tear fluid collection performed at the Norwegian Dry Eye Clinic, as previously outlined [10,58]. In brief, a Schirmer tear test strip (HAAG-STREIT, Essex, UK) was applied to both eyes for 5 min (or more) to produce a minimum combined total of 10 mm of tear volume at room temperature. Then, each Schirmer strip was transferred to 500 µL of 0.1 µm filtered phosphate-buffered saline (Thermo Fisher Scientific, Oslo, Norway) and stored at −80 °C.

### 4.3. Protein Profiling by LC-MS

Initially, in-solution protein digestion was performed for all samples, followed by LC-MS, as outlined formerly [14,21]. In brief, the tryptic peptides (Promega, Madison, WI, USA) were dissolved in 10 µL of 0.1% formic acid (Sigma-Aldrich, Oslo, Norway)/2% acetonitrile (VWR, Oslo, Norway), and 5µL were analysed using an Ultimate 3000 RSLCnano-UHPLC system connected to a Q Exactive mass spectrometer (Thermo Fisher Scientific, Bremen, Germany), and equipped with a nano electrospray ion source. Then, liquid chromatography separation was conducted using an Acclaim PepMap 100 column (Dionex, Sunnyvale, CA, USA). The mass spectrometer was operated in the data-dependent mode to automatically switch between MS and MS/MS acquisition.

### 4.4. LC-MS Data Processing and Statistical Analyses

The LC/MS were searched against the human Uniprot database (20,431 entries), with PEAKS X+ software version 10.5 (Bioinformatics Solutions, Waterloo, ON, Canada). The following parameters were used: digestion enzyme, trypsin; maximum missed cleavage, 1; fragment ion mass error tolerance, 0.05 Da; and parent ion error tolerance, 10.0 ppm. Oxidation of methionine and acetylation of the N-terminus were specified as variable modifications and the maximum number of PTMs was set to 2. A false-discovery rate (FDR) of 1% was applied to the datasets.

For label-free quantification (LFQ) using PEAKS, the following parameters were applied on peptide features: quality ≥ 5, average area ≥ 1 × 10−5, charge: 2–5, peptide ID count per group ≥ 1, detected in at least 3 samples per group; and on protein: significance ≥ 10, fold change ≥ 2, significance method ANOVA with at least 1 peptide. Twenty internal standard proteins were used for normalization. For functional analysis of the proteomics data, DAVID (v 6.7, https://david.ncifcrf.gov (accessed on 12 November 2021)) was used applying a high classification stringency and an enrichment score cut off of 3. Post analytical interpretation of protein functions was performed using the UniProt Knowledgebase (UniProt) (https://www.uniprot.org/ (accessed on 10 January 2022)). Both up- and downregulated proteins were used in the DAVID-, STRING-, and FunRich-analysis [59].

## Figures and Tables

**Table 1:** Upregulated proteins from whole saliva comparing radiated head and neck cancer (HNC) patients, primary Sjögren’s syndrome (pSS) patients, and controls (C) with a fold change of at least two was considered. Proteins found in two different comparisons are shown in bold.

Protein Name	Gene	Comparison	Significance	Fold Change
Aldehyde dehydrogenase dimeric NADP-preferring	*ALDH3A1*	HNC:C	30.3	2.09
Alpha-2-macroglobulin	*A2M*	HNC:pSS	200	3.14
Beta-2-microglobulin	*B2M*	pSS:C	19.35	3.47
BPI fold-containing family B member 2	*BPIFB2*	HNC:pSS	92.23	2.76
Brain acid soluble protein 1	*BASP1*	pSS:C	17.82	3.61
Calumenin	*CALU*	HNC:pSS	101.61	2.37
**Caspase-14**	***CASP14 ***	**HNC:C** **HNC:pSS**	**26.35** **200**	**2.39** **3.09**
Chitinase-3-like protein 2	*CHI3L2*	HNC:pSS	200	3.32
Desmoglein-1	*DSG1*	HNC:pSS	86.71	2.10
Galectin-3-binding protein	*LGALS3BP*	HNC:pSS	89.19	2.42
Gamma-glutamylcyclotransferase	*GGCT*	HNC:pSS	58.86	3.10
Glutathione S-transferase Mu 1	*GSTM1*	HNC:pSS	25.14	8.39
Glyceraldehyde-3-phosphate dehydrogenase	*GAPDH*	HNC:pSS	92.68	2.42
Histone H1.2	*H1-2*	HNC:C	26.44	2.44
Histone H1.3	*H1-3*	HNC:C	22.01	2.16
**Histone H1.4**	***H1-4 ***	**HNC:C** **pSS:C**	**33.38** **27.94**	**2.77** **2.14**
Histone H1.5	*H1-5*	HNC:C	30.78	2.08
**Histone H4**	***H4C1 ***	**HNC:C** **HNC:pSS**	**30.46** **104.63**	**2.28** **2.37**
Integrin alpha-M	*ITGAM*	HNC:pSS	200	2.82
Inter-alpha-trypsin inhibitor heavy chain H1	*ITIH1*	HNC:pSS	25.79	2.54
Kallikrein-1	*KLK1*	HNC:C	17.74	2.01
Kallikrein-6	*KLK6*	HNC:C	26.49	2.03
**Neutrophil collagenase**	***MMP8 ***	**HNC:C** **pSS:C**	**13.65** **19.19**	**2.52** **2.08**
Olfactomedin-4	*OLFM4*	HNC:pSS	27.69	2.25
Perilipin-3	*PLIN3*	HNC:C	16.47	2.82
Proline-rich protein 4	*PRR4*	pSS:C	57.75	6.40
Proteasome subunit beta type-4	*PSMB4*	HNC:C	14.06	2.20
Protein S100-A6	*S100A6*	pSS:C	21.56	2.67
Protein S100-A7	*S100A7*	HNC:pSS	29.11	3.25
Protein S100-A8	*S100A8*	HNC:pSS	200	3.27
**Protein S100-A9**	***S100A9 ***	**HNC:C** **HNC:pSS**	**21.67** **101.13**	**2.20** **2.43**
Prothymosin alpha	*PTMA*	HNC:C	46.53	2.40
Serotransferrin	*TF*	HNC:pSS	55.52	2.06
Serpin B13	*SERPINB13*	HNC:pSS	88.91	2.25
Serum amyloid A-1 protein	*SAA1*	HNC:C	11.58	2.78
SH3 domain-binding glutamic acid-rich-like protein 3	*SH3BGRL3*	HNC:pSS	115.6	2.43
Small proline-rich protein 3	*SPRR3*	HNC:pSS	84.54	2.24
Transcobalamin-1	*TCN1*	HNC:pSS	106.55	2.47
Translationally-controlled tumor protein	*TPT1*	HNC:pSS	112.35	2.60
Vitamin D-binding protein	*GC*	HNC:pSS	103.79	2.22

**Table 2:** Downregulated proteins from whole saliva comparing radiated head and neck cancer (HNC) patients, primary Sjögren’s syndrome (pSS) patients, and controls (C). Proteins found in two different comparisons are shown in bold.

Protein Name	Gene	Comparison	Significance	Fold Change
40S ribosomal protein S6	*RPS6*	pSS:C	11.96	0.15
60S acidic ribosomal protein P2	*RPLP2*	pSS:C	13.74	0.39
60S ribosomal protein L4	*RPL4*	pSS:C	18.81	0.06
**Alpha-amylase 1**	***AMY1 ***	**HNC:C**	**26.68**	**0.21**
		**pSS:C**	**20.27**	**0.15**
Annexin A1	*ANXA1*	HNC:pSS	67.13	0.49
Annexin A2	*ANXA2*	HNC:pSS	105.7	0.39
BPI fold-containing family B member 1	*BPIFB1*	pSS:C	13.9	0.37
Cadherin-1	*CDH1*	pSS:C	13.69	0.39
Calmodulin-like protein 5	*CALML5*	pSS:C	20.17	0.49
Calumenin	*CALU*	pSS:C	10.92	0.43
**Carbonic anhydrase 1**	***CA1 ***	**HNC:C**	**10.62**	**0.04**
		**pSS:C**	**11.59**	**0.07**
**Carboxypeptidase E**	***CPE ***	**HNC:C**	**25.26**	**0.40**
		**pSS:C**	**27.13**	**0.17**
**Cornulin**	***CRNN ***	**HNC:C**	**25.67**	**0.35**
		**pSS:C**	**11.21**	**0.35**
Cystatin-B	*CSTB*	pSS:C	24.08	0.38
Cystatin-C	*CST3*	pSS:C	12.91	0.37
**Cystatin-D**	***CST5 ***	**HNC:C**	**27.49**	**0.38**
		**pSS:C**	**20.87**	**0.32**
**Cystatin-S**	***CST4 ***	**HNC:C**	**64.6**	**0.09**
		**pSS:C**	**29.93**	**0.10**
**Cystatin-SA**	***CST2 ***	**pSS:C**	**31.21**	**0.04**
		**HNC:C**	**26.2**	**0.23**
**Cystatin-SN**	***CST1 ***	**HNC:C**	**49.59**	**0.16**
		**pSS:C**	**41.96**	**0.08**
Desmoplakin	*DSP*	pSS:C	13.7	0.08
EF-hand domain-containing protein D2	*EFHD2*	HNC:pSS	22.24	0.37
Extracellular glycoprotein lacritin	*LACRT*	HNC:pSS	200	0.23
Furin	*FURIN*	pSS:C	29.1	0.30
Galectin-7	*LGALS7*	HNC:C	15.04	0.19
Glutamate dehydrogenase 1 mitochondrial	*GLUD1*	pSS:C	14.06	0.43
Golgi membrane protein 1	*GOLM1*	pSS:C	21.08	0.37
Hemoglobin subunit alpha	*HBA1*	HNC:pSS	200	0.29
Hemoglobin subunit beta	*HBB*	HNC:pSS	93.61	0.46
**Hemoglobin subunit delta**	***HBD ***	**HNC:C**	**11.6**	**0.03**
		**HNC:pSS**	**26.64**	**0.39**
Heterogeneous nuclear ribonucleoprotein A1	*HNRNPA1*	pSS:C	22.89	0.43
Immunoglobulin alpha-2 heavy chain	*N/A*	pSS:C	17.37	0.48
Immunoglobulin heavy constant gamma 1	*IGHG1*	HNC:pSS	78.3	0.46
Immunoglobulin heavy constant gamma 2	*IGHG2*	HNC:C	16.84	0.41
**Immunoglobulin heavy constant gamma 4**	***IGHG4 ***	**HNC:C**	**31.52**	**0.22**
		**pSS:C**	**12.46**	**0.36**
Involucrin	*IVL*	pSS:C	12.97	0.26
Junction plakoglobin	*JUP*	pSS:C	14.57	0.06
Lactotransferrin	*LTF*	pSS:C	37.83	0.10
Mammaglobin-B	*SCGB2A1*	HNC:pSS	200	0.31
Mucin-5B	*MUC5B*	pSS:C	13.84	0.35
Multiple coagulation factor deficiency protein 2	*MCFD2*	HNC:C	22.59	0.33
Neuroblast differentiation-associated protein AHNAK	*AHNAK*	HNC:C	14.22	0.04
Peptidyl-glycine alpha-amidating monooxygenase	*PAM*	pSS:C	18.26	0.14
Peroxiredoxin-1	*PRDX1*	pSS:C	12.08	0.43
Prelamin-A/C	*LMNA*	pSS:C	13.58	0.08
**Prolactin-inducible protein**	***PIP ***	**HNC:C**	**16.76**	**0.39**
		**pSS:C**	**10.49**	**0.43**
Proline-rich protein 27	*PRR27*	HNC:C	10.77	0.48
Proline-rich protein 4	*PRR4*	HNC:pSS	200	0.22
Serpin B5	*SERPINB5*	pSS:C	15.96	0.49
Small proline-rich protein 3	*SPRR3*	pSS:C	33.66	0.37
Soluble calcium-activated nucleotidase 1	*CANT1*	HNC:C	13.12	0.50
Tubulin alpha-4A chain	*TUBA4A*	HNC:C	16.01	0.09
X-ray repair cross-complementing protein 6	*XRCC6*	HNC:C	11.13	0.34
Y-box-binding protein 3	*YBX3*	HNC:C	11.48	0.43

**Table 3:** Functional annotation cluster analysis of saliva.

Comparison	Regulation	Enrichment Score	Enriched Term (Number)	Genes
HCN:C	up	4.59	Histones (5)	*H1-2, H1-3, H1-4, H1-5, H4C9*
HCN:C	up	3.22	Proteases (5)	*CASP14, KLK1, KLK6, MMP8, PSMB4*
HCN:C	down	5.64	Cystatins (4)	*CST1, CST2, CST4, CST5*
pSS:C	down	9.20	Cystatins (6)	*CST1, CST2, CST3, CST4, CST5, CSTB*

HNC: Head and neck cancer patients. pSS: Primary Sjögren’s syndrome patients. C: Healthy controls. Please see Table 1 and Table 2 for full protein names.

**Table 4:** Upregulated proteins in tear fluid from radiated head and neck cancer (HNC) patients compared to the controls (C), with a fold change of at least two considered.

Protein Name	Gene	Significance	Fold Change HNC:C
28 kDa heat- and acid-stable phosphoprotein	*PDAP1*	24.19	2.90
40S ribosomal protein S21	*RPS21*	49.76	2.24
Alpha-1-acid glycoprotein 1	*ORM1*	46.59	2.39
Aminoacylase-1	*ACY1*	12.23	3.42
Apolipoprotein A-I	*APOA1*	54.4	2.34
Apolipoprotein C-III	*APOC3*	36.22	2.53
Apolipoprotein E	*APOE*	25.12	2.21
Beta-2-glycoprotein 1	*APOH*	41.01	3.33
Complement factor I	*CFI*	27.1	2.27
Fibrinogen gamma chain	*FGG*	104.54	2.18
Haptoglobin	*HP*	59.74	2.01
Heterogeneous nuclear ribonucleoprotein U	*HNRNPU*	79.08	3.80
Histone H2B type 1-H	*HIST1H2BH*	46.89	2.66
Immunoglobulin gamma-1 heavy chain	*IGHG1*	81.74	2.67
Immunoglobulin heavy constant gamma 3	*IGHG3*	73.67	2.77
Mucin-like protein 1	*MUCL1*	31.53	5.47
Protein ERGIC-53	*LMAN1*	22.49	2.55
Selenoprotein P	*SELENOP*	33.11	2.84
Vitronectin	*VTN*	66.06	2.28

**Table 5:** Downregulated proteins in tear fluid from radiated head and neck cancer (HNC) patients compared to controls.

Protein Name	Gene	Significance	Fold Change
45 kDa calcium-binding protein	*SDF4 *	62.15	0.36
Acidic leucine-rich nuclear phosphoprotein 32 family member B	*ANP32B *	13.01	0.44
Actin-related protein 2/3 complex subunit 3	*ARPC3 *	41.68	0.39
All-trans-retinol dehydrogenase [NAD(+)] ADH1B	*ADH1B *	23.19	0.5
Alpha-1-acid glycoprotein 2	*ORM2 *	19.26	0.26
Annexin A3	*ANXA3 *	19.31	0.26
Annexin A6	*ANXA6 *	13.92	0.44
Antithrombin-III	*SERPINC1 *	14.47	0.44
Arginase-1	*ARG1 *	17.3	0.14
Barrier-to-autointegration factor	*BANF1 *	12.13	0.44
Basement membrane-specific heparan sulfate proteoglycan core protein	*HSPG2 *	33.77	0.32
Beta-2-microglobulin	*B2M *	34.45	0.29
Bloom syndrome protein	*BLM *	27.56	0.24
Catalase	*CAT *	15.55	0.18
Cathelicidin antimicrobial peptide	*CAMP *	24.11	0.04
Chitinase-3-like protein 2	*CHI3L2 *	55.28	0.35
Clusterin	*CLU *	84.78	0.42
Cystatin-C	*CST3 *	44.94	0.48
Deleted in malignant brain tumors 1 protein	*DMBT1 *	17.29	0.42
DNA damage-binding protein 1	*DDB1 *	90.01	0.49
DnaJ homolog subfamily C member 3	*DNAJC3 *	46.44	0.39
EH domain-containing protein 1	*EHD1 *	20	0.17
Extracellular glycoprotein lacritin	*LACRT *	45.83	0.42
Fructose-bisphosphate aldolase C	*ALDOC *	118.69	0.38
Galectin-3-binding protein	*LGALS3BP *	76.28	0.36
Galectin-7	*LGALS7 *	54.61	0.18
Glucosidase 2 subunit beta	*PRKCSH *	103.78	0.48
Glutaredoxin-1	*GLRX *	97.34	0.48
GMP reductase 2	*GMPR2 *	51.85	0.33
Golgi membrane protein 1	*GOLM1 *	28.43	0.49
Heme-binding protein 1	*HEBP1 *	69.87	0.49
Hepatoma-derived growth factor	*HDGF *	26.23	0.46
Immunoglobulin alpha-2 heavy chain	*N/A*	34.06	0.35
Immunoglobulin kappa constant	*IGKC *	74.47	0.45
Immunoglobulin kappa light chain	*N/A*	34.97	0.34
Immunoglobulin kappa variable 2-24	*IGKV2-24 *	51.08	0.36
Lactotransferrin	*LTF *	10.37	0.32
Lamina-associated polypeptide 2 isoform alpha	*TMPO *	18.11	0.44
Lipocalin-1	*LCN1 *	24.74	0.48
Lymphocyte-specific protein 1	*LSP1 *	10.97	0.27
Macrophage migration inhibitory factor	*MIF *	75.87	0.39
Mesothelin	*MSLN *	10.08	0.48
Metalloproteinase inhibitor 1	*TIMP1 *	51.11	0.46
Methanethiol oxidase	*SELENBP1 *	200	0.47
Monocyte differentiation antigen CD14	*CD14 *	35.97	0.32
Mucin-4	*MUC4 *	24.6	0.2
Multiple coagulation factor deficiency protein 2	*MCFD2 *	23.01	0.47
Myeloperoxidase	*MPO *	27.16	0.27
Neutral alpha-glucosidase AB	*GANAB *	126.44	0.43
Nuclear transport factor 2	*NUTF2 *	43.48	0.38
Nucleobindin-1	*NUCB1 *	89.2	0.38
Nucleobindin-2	*NUCB2 *	44.99	0.42
Opiorphin prepropeptide	*OPRPN *	36.14	0.37
Peptidyl-prolyl cis-trans isomerase B	*PPIB *	44.3	0.47
Phosphatidylethanolamine-binding protein 4	*PEBP4 *	38.46	0.2
Phospholipid transfer protein	*PLTP *	44.98	0.4
Phosphopantothenate--cysteine ligase	*PPCS *	50.18	0.4
Prosaposin	*PSAP *	56.15	0.32
Protein CutA	*CUTA *	104.42	0.47
Ras-related protein Rab-10	*RAB10 *	22.09	0.5
Reticulocalbin-1	*RCN1 *	58.89	0.43
Retinoic acid receptor responder protein 1	*RARRES1 *	77.82	0.35
Secreted frizzled-related protein 1	*SFRP1 *	25.9	0.19
Secretoglobin family 1D member 1	*SCGB1D1 *	45.53	0.39
Septin-2	*SEPTIN2 *	27.53	0.29
Superoxide dismutase [Cu-Zn]	*SOD1 *	200	0.19
Syntaxin-7	*STX7 *	91.29	0.46
Thymosin beta-4	*TMSB4X *	52.28	0.47
Transcobalamin-1	*TCN1 *	47.57	0.38
Translin	*TSN *	45.01	0.48
Vimentin	*VIM *	11.23	0.39
Zinc-alpha-2-glycoprotein	*AZGP1 *	75.58	0.4
Zymogen granule protein 16 homolog B	*ZG16B *	35.01	0.31

**Table 6:** Functional annotation cluster analysis of tears.

Comparison	Regulation	Enrichment Score	Enriched Term (Number)	Genes
HCN:C	down	3.66	EF-hand domain (7)	*EHD1, MCFD2, NUCB1, NUCB2, PRKCSH, RCN1, SDF4*
HCN:C	up	6.72	Secreted/ extracellular (12)	*APOA1, APOC3, APOE, APOH, CFI, FGG, HP, IGHG3, MUCL1, ORM1, SELENOP, VTN*
HCN:C	up	5.13	Lipid-binding (4)	*APOA1, APOC3, APOE, APOH*

HNC: Head- and neck cancer patients. C: Healthy controls. Please see Table 4 and Table 5 for full protein names.

**Table 7:** Clinical characteristics of the radiated patients included in the study.

Patient No.	Age	Sex	Type of Radiotherapy Treatment	Total Radiation Dose (Gy)	Chemotherapy
1	54	M	Primary	68	+
2	75	M	Primary	68	−
3	63	F	Primary	70	+
4	82	F	Primary	68	−
5	61	M	Primary	68	+
6	70	M	Primary	68	+
7	69	F	Primary	68	−
8	58	M	Primary	68	+
9	67	M	Primary	68	+
10	59	M	Primary	68	−
11	53	M	Primary	68	+
12	64	M	Primary	68	+
13	57	M	Primary	68	+
14	68	M	Primary	68	+
15	73	M	Postoperative	56	−
16	66	F	Postoperative	66	−
17	65	F	Postoperative	60	−
18	73	F	Postoperative	66	−
19	71	F	Postoperative	60	−
20	66	F	Postoperative	66	−
21	51	F	Postoperative	66	−
22	58	M	Postoperative	60	−
23	41	F	Postoperative	60	+
24	82	M	Postoperative	60	−
25	51	F	Postoperative	60	+
26	65	F	Postoperative	66	−
27	58	M	Postoperative	60	−
28	60	F	Postoperative	50	−
29	82	M	Postoperative	60	−

M: male; F: female.

**Table 8:** Clinical characteristics of pSS patients included in the study.

Patient No.	Age	Sex	Anti- SSA *	Anti- SSB *	Focus Score **	Schirmer Test ***	Saliva Secretion ****	Dry Mouth	Dry Eyes
1	64	F	+	−	NT	−	+	+	+
2	68	F	+	+	1	+	+	+	+
3	72	F	+	+	NT	NT	+	+	+
4	71	F	+	−	NT	+	−	+	+
5	57	F	+	+	NT	+	+	+	+
6	57	F	+	−	0	+	+	−	+
7	73	F	+	−	<1	+	+	+	+
8	65	F	+	−	<1	+	+	+	+
9	56	F	+	−	1	+	+	+	+
10	68	F	+	+	NT	−	+	+	+
11	75	F	+	+	NT	+	−	+	+
12	50	F	+	+	NT	NT	+	+	+
13	60	F	+	+	2	+	−	+	−
14	51	F	+	−	8	+	−	−	−

F: female; NT: not tested. * Autoantibody production was assessed by ELISA. ** Values are the number of focal infiltrates/4 mm^2^ tissue area containing >50 mononuclear cells. *** Values are in mm/5 min; normal flow > 5 mm/5 min. ‘+’ indicates dryness and tear secretion ≤5 mm/5 min. **** Values are in mL/15 min; normal flow > 1.5 mL/15 min. ‘+’ indicates dryness and unstimulated whole saliva secretion ≤ 1.5 mL/15 min.

## Data Availability

The datasets generated and/or analysed during the current study are not publicly available due to ethical restrictions enforced by the research and medical institutions under license for the current study. Data are, however, available from the authors upon reasonable request and with permission of the Regional Medical Ethical Committee of South-East Norway, the University of Oslo and Oslo University Hospital.
